# Interaction between an edge dislocation and a circular elastic inhomogeneity with Steigmann–Ogden interface

**DOI:** 10.1177/10812865231166081

**Published:** 2023-04-26

**Authors:** Xu Wang, Peter Schiavone

**Affiliations:** School of Mechanical and Power Engineering, East China University of Science and Technology, Shanghai, China; Department of Mechanical Engineering, University of Alberta, Edmonton, AB, Canada

**Keywords:** Steigmann-Ogden interface, circular inhomogeneity, edge dislocation, analytic continuation, analytical solution

## Abstract

We propose an effective method for the solution of the plane problem of an edge dislocation in the vicinity of a circular inhomogeneity with Steigmann–Ogden interface. Using analytic continuation, the pair of analytic functions defined in the infinite matrix surrounding the inhomogeneity can be expressed in terms of the pair of analytic functions defined inside the circular inhomogeneity. Once the two analytic functions defined in the circular inhomogeneity are expanded in Taylor series with unknown complex coefficients, the Steigmann–Ogden interface condition can be written explicitly in complex form. Consequently, all of the complex coefficients appearing in the Taylor series can be uniquely determined so that the two pairs of analytic functions are then completely determined. An explicit and general expression of the image force acting on the edge dislocation is derived using the Peach–Koehler formula.

## 1. Introduction

The first continuum-based surface/interface model was established by Gurtin, Murdoch and co-workers [1–3]. This model, now commonly referred to in the literature as the “Gurtin-Murdoch model”, was recently clarified by Ru [4]. In the Gurtin–Murdoch model, the surface is treated as an elastic membrane of vanishing thickness adhering to the bulk without slipping. Consequently, the surface is resistant only to stretching (and not bending) rendering it incapable of supporting a compressive stress state. Steigmann and Ogden [5,6] improved the Gurtin–Murdoch theory by endowing the surface model with both stretching and flexural resistance. In other words, the surface energy in the Steigmann–Ogden model is a function of both the surface strain tensor and the surface curvature tensor. The Steigmann–Ogden model has been adopted in the analysis of a range of problems including those dealing with nanoscale composites [7–12].

In this paper, we study an edge dislocation interacting with a circular elastic inhomogeneity incorporating a Steigmann–Ogden interface. The resulting boundary value problem poses considerable challenges due to the complexity of the boundary conditions on the Steigmann–Ogden circular interface [7,9,10]. This perhaps explains why the solution of this interaction problem remains absent from the literature. Despite this complexity, we propose a simple and effective method which permits an analytical solution to this plane problem. Using analytic continuation [13–15] to enforce the condition of continuity of displacements across the Steigmann–Ogden interface, the pair of analytic functions defined in the surrounding matrix can be expressed in terms of the pair of analytic functions defined inside the circular inhomogeneity. Once the two analytic functions in the circular inhomogeneity are expressed in standard Taylor series with unknown complex coefficients, the complex form of the Steigmann–Ogden interface condition (specifically the relation between the jumps in interfacial normal and tangential tractions and the interfacial normal and tangential displacements and their tangential derivatives, see [7,10]) can be expressed explicitly. Consequently, all of the complex coefficients appearing in the Taylor series can be uniquely determined so that the pair of analytic functions defined in the matrix is then completely determined. Using the Peach–Koehler formula [16], we also establish the image force acting on the edge dislocation. The resulting explicit expression for the image force is quite general in the sense that both components of the Burgers vector can be nonzero. When the surface effect is neglected for a perfect interface, our result for the image force on a gliding edge dislocation simply recovers the classical result by Dundurs [16].

## 2. Complex variable formulation for plane isotropic elasticity

A Cartesian coordinate system $\left\{x_{i}\right\}\;\;(i=1,2,3)$ is established. For the in-plane deformations of an isotropic elastic material, the three in-plane stresses $\left(\sigma_{11},\;\sigma_{22},\;\sigma_{12}\right)$ , two in-plane displacements $\left(u_{1},\;u_{2}\right)$ and two stress functions $\left(\phi_{1},\;\phi_{2}\right)$ are given in terms of two analytic functions φ(z) and ψ(z) of the complex variable $z=x_{1}+ix_{2}$ as [17]

(1) $$\begin{array}{c} \sigma_{11}+\sigma_{22}=2\left[\varphi \prime (z)+\bar{\varphi \prime (z)}\right],\\ \sigma_{22}-\sigma_{11}+2i\sigma_{12}=2\left[\bar{z}\varphi \prime \prime (z)+\psi \prime (z)\right], \end{array}$$

(2) $$\begin{array}{c} 2\mu (u_{1}+iu_{2})=\kappa \varphi (z)-z\bar{\varphi \prime (z)}-\bar{\psi (z)},\\ \phi_{1}+i\phi_{2}=i\left[\varphi (z)+z\bar{\varphi \prime (z)}+\bar{\psi (z)}\right], \end{array}$$

where κ=3−4ν for plane strain, which is assumed is this paper, and κ=(3−ν)/(1+ν) for plane stress; *µ* and ν(0≤ν≤1/2) are the shear modulus and Poisson’s ratio, respectively. In addition, the stresses are related to the two stress functions through [18]

(3) $$\begin{array}{c} \sigma_{11}=-\phi_{1,2},\;\sigma_{12}=\phi_{1,1},\\ \sigma_{21}=-\phi_{2,2},\;\sigma_{22}=\phi_{2,1}. \end{array}$$

## 3. An edge dislocation near a circular inhomogeneity with Steigmann-Ogden interface

Consider a domain in ℜ^2^, infinite in extent, containing a circular inhomogeneity of radius *R* with its center at the origin of the Cartesian coordinate system. Let $S_{1}$ and $S_{2}$ denote the inhomogeneity and the matrix, respectively, which are bonded through the Steigmann–Ogden circular interface *L*. An edge dislocation with Burgers vector $\left(b_{1},b_{2}\right)$ is applied at (ξ,0) on the $x_{1}$ -axis in the matrix. In what follows, the subscripts 1 and 2 (or the superscripts (1) and (2)) are used to identify the respective quantities in $S_{1}$ and $S_{2}$ .

According to Zemlyanova and Mogilevskaya [7], the boundary conditions on the Steigmann–Ogden circular interface can be expressed in polar coordinates (r,θ) and in complex form as follows

(4) $$\begin{array}{c} u_{r}^{\left(1\right)}+iu_{\theta }^{\left(1\right)}=u_{r}^{\left(2\right)}+iu_{\theta }^{\left(2\right)},\\ \sigma_{\mathrm{rr}}^{\left(1\right)}+i\sigma_{r\theta }^{\left(1\right)}-(\sigma_{\mathrm{rr}}^{\left(2\right)}+i\sigma_{r\theta }^{\left(2\right)})=-\frac{\sigma_{0}}{R}-\frac{J_{0}}{R^{2}}\left[u_{r}^{\left(1\right)}-i\frac{\partial^{2}}{\partial \theta^{2}}u_{\theta }^{\left(1\right)}-i\frac{\partial }{\partial \theta }(u_{r}^{\left(1\right)}+iu_{\theta }^{\left(1\right)})\right]\\ \;+\left(\frac{\sigma_{0}}{R^{2}}-\frac{K_{0}}{R^{4}}\frac{\partial^{2}}{\partial \theta^{2}}\right)\left[\frac{\partial^{2}}{\partial \theta^{2}}u_{r}^{\left(1\right)}-iu_{\theta }^{\left(1\right)}+i\frac{\partial }{\partial \theta }(u_{r}^{\left(1\right)}+iu_{\theta }^{\left(1\right)})\right],\;\;\left|z\right|=R, \end{array}$$

where

(5) $$J_{0}=2\mu_{0}+\lambda_{0}\geq 0,\;\;K_{0}=2\chi +\zeta \geq 0.$$

In Equations (4) and (5), $\sigma_{0}$ is the residual surface tension, $\lambda_{0}$ and $\mu_{0}$ are the two surface Lame parameters, while *χ* and *ζ* are two bending stiffness parameters. Using Equation (2)_1_, the continuity of displacements across the circular interface in Equation (4)_1_ can be expressed as

(6) $$\Gamma \kappa_{1}\varphi_{1}(z)-\Gamma \left[\bar{\psi_{1}(z)}+z\bar{{\varphi \prime }_{1}(z)}\right]=\kappa_{2}\varphi_{2}(z)-\left[\bar{\psi_{2}(z)}+z\bar{{\varphi \prime }_{2}(z)}\right],\;\;\left|z\right|=R,$$

where

(7) $$\Gamma =\frac{\mu_{2}}{\mu_{1}}.$$

Using analytic continuation [13–15], Equation (6) can be further written in the form

(8) $$\begin{array}{c} \Gamma \kappa_{1}\varphi_{1}^{+}(z)+z\bar{\varphi }{\prime_{2}}^{+}\left(\frac{R^{2}}{z}\right)+{\bar{\psi }}_{2}^{+}\left(\frac{R^{2}}{z}\right)-\kappa_{2}A\ln(z-\xi )\\ -A\ln\frac{z-R^{2}/\xi }{z}+\frac{R^{2}(R^{2}-\xi^{2})}{\xi^{3}}\frac{\bar{A}}{z-R^{2}/\xi }-\Gamma \bar{{\varphi \prime }_{1}(0)}z\\ =\kappa_{2}\varphi_{2}^{-}(z)+\Gamma \left[z{\bar{\varphi }}_{1}^{\prime -}\left(\frac{R^{2}}{z}\right)+{\bar{\psi }}_{1}^{-}\left(\frac{R^{2}}{z}\right)\right]-\kappa_{2}A\ln(z-\xi )\\ -A\ln\frac{z-R^{2}/\xi }{z}+\frac{R^{2}(R^{2}-\xi^{2})}{\xi^{3}}\frac{\bar{A}}{z-R^{2}/\xi }-\Gamma \bar{{\varphi \prime }_{1}(0)}z,\;\;\left|z\right|=R, \end{array}$$

where

(9) $$A=\frac{\mu_{2}(b_{2}-ib_{1})}{\pi (\kappa_{2}+1)}.$$

***Remark*.** In the proofs, there are mistakes in the writing of the second term “ $z{\bar{\varphi }}_{2}^{\prime }+\left(\frac{R^{2}}{z}\right)$ ”on the left-hand side of Equation (8) and the second term “ $\Gamma \left[z{\bar{\varphi }}_{1}^{\prime -}\left(\frac{R^{2}}{z}\right)+{\bar{\psi }}_{1}^{-}\left(\frac{R^{2}}{z}\right)\right]$ ”on the right-hand side of Equation (8). Please write the plus “+” and minus “–” into superscripts.

(10) $$\begin{array}{c} \varphi_{2}(z)=A\ln(z-\xi )+\frac{\Gamma }{\kappa_{2}}\bar{{\varphi \prime }_{1}(0)}z-\frac{\Gamma }{\kappa_{2}}\left[z{\bar{\varphi }\prime }_{1}\left(\frac{R^{2}}{z}\right)+{\bar{\psi }}_{1}\left(\frac{R^{2}}{z}\right)\right]+\frac{1}{\kappa_{2}}\left[A\ln\frac{z-R^{2}/\xi }{z}-\frac{R^{2}(R^{2}-\xi^{2})}{\xi^{3}}\frac{\bar{A}}{z-R^{2}/\xi }\right],\\ \psi_{2}(z)+\frac{R^{2}}{z}{\varphi \prime }_{2}(z)=\bar{A}\ln(z-\xi )+\frac{A(R^{2}-\xi^{2})}{\xi (z-\xi )}-\Gamma \kappa_{1}{\bar{\varphi }}_{1}\left(\frac{R^{2}}{z}\right)+\kappa_{2}\bar{A}\ln\frac{z-R^{2}/\xi }{z}+\frac{R^{2}\Gamma {\varphi \prime }_{1}(0)}{z}. \end{array}$$

Once $\varphi_{1}(z)$ and $\psi_{1}(z)$ defined in the inhomogeneity are known, $\varphi_{2}(z)$ and $\psi_{2}(z)$ defined in the matrix can be determined from Equation (10). Using Equation (2)_2_, the jumps in the two stress functions across the circular interface can be expressed as

(11) $$\phi_{2}^{\left(1\right)}-i\phi_{1}^{\left(1\right)}-(\phi_{2}^{\left(2\right)}-i\phi_{1}^{\left(2\right)})=\varphi_{1}^{+}(z)+z\bar{\varphi }\prime_{1}^{-}\left(\frac{R^{2}}{z}\right)+{\bar{\psi }}_{1}^{-}\left(\frac{R^{2}}{z}\right)-\varphi_{2}^{-}(z)-z\bar{\varphi }\prime_{2}^{+}\left(\frac{R^{2}}{z}\right)-{\bar{\psi }}_{2}^{+}\left(\frac{R^{2}}{z}\right),\;\;\left|z\right|=R.$$

With the aid of Equation (10), we can rewrite Equation (11) as the following

(12) $$\begin{array}{c} \phi_{2}^{\left(1\right)}-i\phi_{1}^{\left(1\right)}-(\phi_{2}^{\left(2\right)}-i\phi_{1}^{\left(2\right)})=(\Gamma \kappa_{1}+1)\varphi_{1}^{+}(z)+\frac{\Gamma +\kappa_{2}}{\kappa_{2}}\left[z\bar{\varphi }\prime_{1}^{-}\left(\frac{R^{2}}{z}\right)+{\bar{\psi }}_{1}^{-}\left(\frac{R^{2}}{z}\right)\right]\\ -(\kappa_{2}+1)A\ln(z-\xi )-\frac{\kappa_{2}+1}{\kappa_{2}}\left[A\ln\frac{z-R^{2}/\xi }{z}-\frac{R^{2}(R^{2}-\xi^{2})}{\xi^{3}}\frac{\bar{A}}{z-R^{2}/\xi }\right]-\frac{\Gamma (\kappa_{2}+1)}{\kappa_{2}}\bar{{\varphi \prime }_{1}(0)}z,\;\;\left|z\right|=R. \end{array}$$

The pair of analytic functions $\varphi_{1}(z)$ and $\psi_{1}(z)$ defined in the circular inhomogeneity can be expanded in convergent Taylor series as follows:

(13) $$\varphi_{1}(z)=\sum_{n=1}^{+\infty }A_{n}z^{n},\;\;\psi_{1}(z)=\sum_{n=1}^{+\infty }B_{n}z^{n},$$

where $A_{n},\;\;B_{n}\;\;(n=1,2,\cdots ,+\infty )$ are unknown complex constants to be determined.

Inserting Equation (13) into Equation (12) and expanding the three functions $\ln(z-\xi ),\;\;\ln\frac{z-R^{2}/\xi }{z}$ and $\frac{1}{z-R^{2}/\xi }$ in Equation (12) into convergent Laurent series, we arrive at

(14) $$\begin{array}{c} \phi_{2}^{\left(1\right)}-i\phi_{1}^{\left(1\right)}-(\phi_{2}^{\left(2\right)}-i\phi_{1}^{\left(2\right)})=\left[A_{1}(\Gamma \kappa_{1}+1)+{\bar{A}}_{1}(1-\Gamma )\right]z+(\Gamma \kappa_{1}+1)A_{2}z^{2}\\ +\sum_{n=3}^{+\infty }(\Gamma \kappa_{1}+1)A_{n}z^{n}+\sum_{n=1}^{+\infty }\frac{R^{2(n+1)}(\Gamma +\kappa_{2})}{\kappa_{2}}\left[(n+2){\bar{A}}_{n+2}+R^{-2}{\bar{B}}_{n}\right]\frac{1}{z^{n}}\\ +A(\kappa_{2}+1)\sum_{n=1}^{+\infty }\frac{z^{n}}{n\xi^{n}}-\frac{\kappa_{2}+1}{\kappa_{2}}\left[-A\sum_{n=1}^{+\infty }\frac{R^{2n}}{n\xi^{n}}\frac{1}{z^{n}}-\frac{\bar{A}R^{2}(R^{2}-\xi^{2})}{\xi^{3}}\sum_{n=0}^{+\infty }\frac{R^{2n}}{\xi^{n}}\frac{1}{z^{n+1}}\right],\;\;\left|z\right|=R. \end{array}$$

Using Equation (14), the jumps in normal and tangential tractions across the circular interface, which are simply the left-hand side of the interface condition in Equation (4)_2_, are given explicitly by

(15) $$\begin{array}{c} \sigma_{\mathrm{rr}}^{\left(1\right)}+i\sigma_{r\theta }^{\left(1\right)}-(\sigma_{\mathrm{rr}}^{\left(2\right)}+i\sigma_{r\theta }^{\left(2\right)})=\frac{d}{\mathrm{dz}}\left[\phi_{2}^{\left(1\right)}-i\phi_{1}^{\left(1\right)}-(\phi_{2}^{\left(2\right)}-i\phi_{1}^{\left(2\right)})\right]\\ =A_{1}(\Gamma \kappa_{1}+1)+{\bar{A}}_{1}(1-\Gamma )+\frac{A(\kappa_{2}+1)}{\xi }+\left[2(\Gamma \kappa_{1}+1)A_{2}+\frac{A(\kappa_{2}+1)}{\xi^{2}}\right]z\\ +\sum_{n=2}^{+\infty }\left\{(n+1)(\Gamma \kappa_{1}+1)A_{n+1}+\frac{A(\kappa_{2}+1)}{\xi^{n+1}}\right\}z^{n}\\ +\sum_{n=2}^{+\infty }\left\{-\frac{R^{2n}(n-1)(\Gamma +\kappa_{2})}{\kappa_{2}}\left[(n+1){\bar{A}}_{n+1}+R^{-2}{\bar{B}}_{n-1}\right]-\frac{\kappa_{2}+1}{\kappa_{2}}\left[\frac{AR^{2(n-1)}}{\xi^{\left(n-1\right)}}+\frac{\bar{A}R^{2(n-1)}(R^{2}-\xi^{2})(n-1)}{\xi^{n+1}}\right]\right\}\frac{1}{z^{n}},\\ \left|z\right|=R. \end{array}$$

Using Equations (2)_1_ and (13), the interfacial normal and tangential displacements are given by

(16) $$\begin{array}{c} 2\mu_{1}(u_{r}+iu_{\theta })=R(\kappa_{1}A_{1}-{\bar{A}}_{1})+R\kappa_{1}A_{2}z-2R^{3}{\bar{A}}_{2}\frac{1}{z}+\sum_{n=2}^{+\infty }\kappa_{1}RA_{n+1}z^{n}\\ -\sum_{n=2}^{+\infty }R^{2n+1}\left[(n+1){\bar{A}}_{n+1}+R^{-2}{\bar{B}}_{n-1}\right]\frac{1}{z^{n}},\;\;\left|z\right|=R. \end{array}$$

By considering Equation (16), the right-hand side of the interface condition in Equation (4)_2_ can also be written explicitly as

(17) $$\begin{array}{c} -\frac{\sigma_{0}}{R}-\frac{J_{0}}{R^{2}}\left[u_{r}^{\left(1\right)}-i\frac{\partial^{2}}{\partial \theta^{2}}u_{\theta }^{\left(1\right)}-i\frac{\partial }{\partial \theta }(u_{r}^{\left(1\right)}+iu_{\theta }^{\left(1\right)})\right]+\left(\frac{\sigma_{0}}{R^{2}}-\frac{K_{0}}{R^{4}}\frac{\partial^{2}}{\partial \theta^{2}}\right)\left[\frac{\partial^{2}}{\partial \theta^{2}}u_{r}^{\left(1\right)}-iu_{\theta }^{\left(1\right)}+i\frac{\partial }{\partial \theta }(u_{r}^{\left(1\right)}+iu_{\theta }^{\left(1\right)})\right]\\ =-\frac{\sigma_{0}}{R}-\frac{1}{4\mu_{1}R}\left[J_{0}(\kappa_{1}-1)(A_{1}+{\bar{A}}_{1})+\sigma_{0}(\kappa_{1}+1)(A_{1}-{\bar{A}}_{1})\right]-\frac{\kappa_{1}(J_{0}+\sigma_{0}+R^{-2}K_{0})}{\mu_{1}R}A_{2}z\\ -\sum_{n=2}^{+\infty }\left[\frac{{\left(n+1\right)}^{2}\left[(\kappa_{1}-1+n)J_{0}+(\kappa_{1}+1-n)(\sigma_{0}+n^{2}R^{-2}K_{0})\right]}{4\mu_{1}R}A_{n+1}+\frac{\left(n^{2}-1)(J_{0}-\sigma_{0}-n^{2}R^{-2}K_{0}\right)}{4\mu_{1}R}R^{-2}B_{n-1}\right]z^{n}\\ +\sum_{n=2}^{+\infty }\left[\frac{(n^{2}-1)\left[(\kappa_{1}-1+n)J_{0}-(\kappa_{1}+1-n)(\sigma_{0}+n^{2}R^{-2}K_{0})\right]}{4\mu_{1}R}{\bar{A}}_{n+1}+\frac{{\left(n-1\right)}^{2}(J_{0}+\sigma_{0}+n^{2}R^{-2}K_{0})}{4\mu_{1}R}R^{-2}{\bar{B}}_{n-1}\right]\frac{R^{2n}}{z^{n}},\\ \left|z\right|=R. \end{array}$$

In deriving Equation (17), we first consider the term in $z^{0}$ in Equation (16), then the terms in z and $z^{-1}$ in Equation (16), and third the terms in $z^{n}$ and $z^{-n}$ ( n≥2 ) in Equation (16). It is clear that Equation (17) does not contain a term in $z^{-1}$ . In view of Equations (4)_2_, (15) and (17), all of the complex constants $A_{n},\;B_{n}\;\;(n=1,2,\cdots ,+\infty )$ can be uniquely determined as follows:

(18) $$A_{1}=-\frac{\frac{\sigma_{0}}{R}+\frac{\mu_{2}b_{2}}{\xi \pi }}{\Gamma (\kappa_{1}-1)+2+\frac{J_{0}(\kappa_{1}-1)}{2\mu_{1}R}}+\frac{i\mu_{2}b_{1}}{\xi \pi (\kappa_{1}+1)\left(\Gamma +\frac{\sigma_{0}}{2\mu_{1}R}\right)},$$

(19) $$A_{2}=-\frac{A(\kappa_{2}+1)}{\xi^{2}\left[2(\Gamma \kappa_{1}+1)+\frac{\kappa_{1}(J_{0}+\sigma_{0}+R^{-2}K_{0})}{\mu_{1}R}\right]},$$

(20) $$\begin{array}{c} A_{n+1}=\frac{(\kappa_{2}+1)\left(\begin{array}{c} -\frac{A}{\xi^{n+1}}\left[\frac{\Gamma +\kappa_{2}}{\kappa_{2}}+\frac{\left(n-1)(J_{0}+\sigma_{0}+n^{2}R^{-2}K_{0}\right)}{4\mu_{1}R}\right]\\ +\frac{\left(n+1)(J_{0}-\sigma_{0}-n^{2}R^{-2}K_{0}\right)}{4\kappa_{2}\mu_{1}R}\left[\frac{\bar{A}}{R^{2}\xi^{n-1}}+\frac{A(1-R^{-2}\xi^{2})(n-1)}{\xi^{n+1}}\right] \end{array}\right)}{\Delta_{n}(n+1)},\\ R^{-2}B_{n-1}=\frac{(\kappa_{2}+1)\left(\begin{array}{c} \frac{A(n-1)}{\xi^{n+1}}\left[\frac{\Gamma +\kappa_{2}}{\kappa_{2}}+\frac{\left(\kappa_{1}-1+n)J_{0}-(\kappa_{1}+1-n)(\sigma_{0}+n^{2}R^{-2}K_{0}\right)}{4\mu_{1}R}\right]\\ -\frac{1}{\kappa_{2}}\left[\Gamma \kappa_{1}+1+\frac{(n+1)\left[(\kappa_{1}-1+n)J_{0}+(\kappa_{1}+1-n)(\sigma_{0}+n^{2}R^{-2}K_{0})\right]}{4\mu_{1}R}\right]\\ \times \left[\frac{\bar{A}}{R^{2}\xi^{n-1}}+\frac{A(1-R^{-2}\xi^{2})(n-1)}{\xi^{n+1}}\right] \end{array}\right)}{\Delta_{n}(n-1)},\\ n=2,3,\cdots ,+\infty , \end{array}$$

where

(21) $$\begin{array}{c} \Delta_{n}=\left[\Gamma \kappa_{1}+1+\frac{(n+1)\left[(\kappa_{1}-1+n)J_{0}+(\kappa_{1}+1-n)(\sigma_{0}+n^{2}R^{-2}K_{0})\right]}{4\mu_{1}R}\right]\\ \times \left[\frac{\Gamma +\kappa_{2}}{\kappa_{2}}+\frac{\left(n-1)(J_{0}+\sigma_{0}+n^{2}R^{-2}K_{0}\right)}{4\mu_{1}R}\right]-\frac{\left(n^{2}-1)(J_{0}-\sigma_{0}-n^{2}R^{-2}K_{0}\right)}{4\mu_{1}R}\\ \times \left[\frac{\Gamma +\kappa_{2}}{\kappa_{2}}+\frac{\left(\kappa_{1}-1+n)J_{0}-(\kappa_{1}+1-n)(\sigma_{0}+n^{2}R^{-2}K_{0}\right)}{4\mu_{1}R}\right]. \end{array}$$

***Remark.*** Each of Re{A1},Im{A1} and $A_{2}$ is determined by solving a single linear algebraic equation; the two complex constants $A_{n+1}$ and $B_{n-1}$ with n≥2 are determined by solving two coupled linear algebraic equations.

It is further deduced from Equation (18) that the rigid-body rotation at the center of the circular inhomogeneity is

(22) $$\varpi_{21}=\frac{1}{2}(u_{2,1}-u_{1,2})=\frac{b_{1}}{2\xi \pi \left(1+\frac{\sigma_{0}}{2\mu_{2}R}\right)},$$

and the average mean stress within the circular inhomogeneity is

(23) $$<\sigma_{11}+\sigma_{22}>=-\frac{4\left(\frac{\sigma_{0}}{R}+\frac{\mu_{2}b_{2}}{\xi \pi }\right)}{2+(\kappa_{1}-1)\left(\Gamma +\frac{J_{0}}{2\mu_{1}R}\right)},$$

where <*> denotes the average. The average mean stress within the circular inhomogeneity can be nullified when

(24) $$\frac{\xi }{R}=-\frac{\mu_{2}b_{2}}{\pi \sigma_{0}}\;\left(\left|\frac{\xi }{R}\right|>1\right).$$

By substituting Equation (13) into Equation (10), the pair of analytic functions $\varphi_{2}(z)$ and $\psi_{2}(z)$ defined in the matrix can be given explicitly by

(25) $$\begin{array}{c} \varphi_{2}(z)=A\ln(z-\xi )+\frac{1}{\kappa_{2}}\left[A\ln\frac{z-R^{2}/\xi }{z}-\frac{R^{2}(R^{2}-\xi^{2})}{\xi^{3}}\frac{\bar{A}}{z-R^{2}/\xi }\right]-\frac{\Gamma }{\kappa_{2}}\sum_{n=1}^{+\infty }R^{2(n+1)}\left[(n+2){\bar{A}}_{n+2}+R^{-2}{\bar{B}}_{n}\right]\frac{1}{z^{n}},\\ \psi_{2}(z)=\bar{A}\ln(z-\xi )-\frac{A\xi }{z-\xi }+\kappa_{2}\bar{A}\ln\frac{z-R^{2}/\xi }{z}+\frac{1}{\kappa_{2}}\left[\frac{\bar{A}(R^{2}-\xi^{2})}{\xi }-A\xi \right]\frac{1}{z-R^{2}/\xi }\\ -\frac{\bar{A}R^{2}(R^{2}-\xi^{2})}{\kappa_{2}\xi^{2}}\frac{1}{{\left(z-R^{2}/\xi \right)}^{2}}+\left[\left(\frac{R^{2}}{\xi }+\frac{\xi }{\kappa_{2}}\right)A+\frac{\bar{A}(\xi^{2}-R^{2})}{\kappa_{2}\xi }+R^{2}\Gamma A_{1}-R^{2}\Gamma \kappa_{1}{\bar{A}}_{1}\right]\frac{1}{z}+\left(\frac{R^{2}A}{\kappa_{2}}-\Gamma \kappa_{1}R^{4}{\bar{A}}_{2}\right)\frac{1}{z^{2}}\\ -\Gamma \sum_{n=3}^{+\infty }R^{2n}\left[\left(\frac{n(n-2)}{\kappa_{2}}+\kappa_{1}\right){\bar{A}}_{n}+\frac{n-2}{\kappa_{2}}R^{-2}{\bar{B}}_{n-2}\right]\frac{1}{z^{n}}. \end{array}$$

Using the Peach-Koehler formula [16], the image force acting on the edge dislocations is given by

(26) F1−iF2=2(b2+ib1)Re{φ′R(ξ)}+(b2−ib1)[ξφ″R(ξ)+ψ′R(ξ)],

where $F_{1}$ and $F_{2}$ are, respectively, the image force components along the $x_{1}$ and $x_{2}$ -directions, and $\varphi \prime_{R}(\xi )$ , $\xi {\varphi \prime \prime }_{R}(\xi )+\psi \prime_{R}(\xi )$ are given by

(27) Re{φ′R(ξ)}=Γκ2∑n=2+∞R2nξn(n−1)Re{(n+1)An+1+R−2Bn−1},ξφ″R(ξ)+ψ′R(ξ)=A(ξ2−R2)+A¯ξ2κ2ξ(ξ2−R2)+κ2R2A¯ξ(ξ2−R2)−[A(R2ξ+ξκ2)+A¯(ξ2−R2)κ2ξ+R2Γ(A1−κ1A¯1)]1ξ2−2(R2Aκ2−Γκ1R4A¯2)1ξ3+∑n=2+∞R2nΓξn+2{(n−1)[R2(n+1)−ξ2n]κ2[(n+1)A¯n+1+R−2B¯n−1]+(n+1)R2κ1A¯n+1}.

The term $(n+1)A_{n+1}+R^{-2}B_{n-1}$ in Equation (27) can be determined explicitly as

(28) $$\begin{array}{c} (n+1)A_{n+1}+R^{-2}B_{n-1}\\ =\frac{(\kappa_{2}+1)\left(\begin{array}{c} \frac{A}{\xi^{n+1}}\frac{\kappa_{1}(J_{0}-\sigma_{0}-n^{2}R^{-2}K_{0})}{4\mu_{1}R}\\ -\frac{1}{\kappa_{2}}\left[\Gamma \kappa_{1}+1+\frac{\left(n+1)\kappa_{1}(J_{0}+\sigma_{0}+n^{2}R^{-2}K_{0}\right)}{4\mu_{1}R}\right]\left[\frac{\bar{A}}{R^{2}\xi^{n-1}(n-1)}+\frac{A(1-R^{-2}\xi^{2})}{\xi^{n+1}}\right] \end{array}\right)}{\Delta_{n}}. \end{array}$$

It is clear from Equation (26) that $F_{2}\neq 0$ when $b_{1}\cdot b_{2}\neq 0$ . The image force on a gliding edge dislocation with $b_{2}=0$ is

(29) $$\begin{array}{c} \frac{\pi R}{\mu_{2}b_{1}^{2}}F_{1}=\frac{\kappa_{2}^{2}+1}{\kappa_{2}(\kappa_{2}+1)\lambda (\lambda^{2}-1)}+\frac{1}{\lambda^{3}}\left[\frac{\kappa_{2}+3}{\kappa_{2}(\kappa_{2}+1)}-\frac{\Gamma }{\Gamma +\frac{\sigma_{0}}{2\mu_{1}R}}\right]-\frac{1}{\lambda^{5}}\frac{2\Gamma \kappa_{1}}{2(\Gamma \kappa_{1}+1)+\frac{\kappa_{1}(J_{0}+\sigma_{0}+R^{-2}K_{0})}{\mu_{1}R}}\\ -\frac{\Gamma }{\kappa_{2}^{2}}\sum_{n=2}^{+\infty }\frac{1}{\Delta_{n}}\left(\begin{array}{c} \frac{1}{\lambda^{2n+3}}\left[\begin{array}{c} \kappa_{1}\kappa_{2}(\Gamma +\kappa_{2})+(n^{2}-1)(\Gamma \kappa_{1}+1)-\lambda^{2}n(n-1)(\Gamma \kappa_{1}+1)\\ +\frac{\kappa_{1}(n-1)}{4\mu_{1}R}\left[\begin{array}{c} (n+1-\kappa_{2})(n+1-\kappa_{2}-\lambda^{2}n)J_{0}\\ +(n+1+\kappa_{2})(n+1+\kappa_{2}-\lambda^{2}n)(\sigma_{0}+n^{2}R^{-2}K_{0}) \end{array}\right] \end{array}\right]\\ -\frac{n}{\lambda^{2n+1}}\left[\begin{array}{c} \left(\Gamma \kappa_{1}+1)(n+1-\lambda^{2}n\right)\\ +\frac{\kappa_{1}(n+1)}{4\mu_{1}R}\left[(n+1-\kappa_{2}-\lambda^{2}n)J_{0}+(n+1+\kappa_{2}-\lambda^{2}n)(\sigma_{0}+n^{2}R^{-2}K_{0})\right] \end{array}\right] \end{array}\right),\\ F_{2}=0, \end{array}$$

where

(30) $$\lambda =\frac{\xi }{R}.$$

As a quick check, when $\sigma_{0}=J_{0}=K_{0}=0$ for a perfect circular interface, Equation (29) reduces to

(31) $$\frac{\pi R}{\mu_{2}b_{1}^{2}}F_{1}=\left[\frac{\kappa_{2}^{2}+1}{\kappa_{2}(\kappa_{2}+1)}-\frac{\Gamma \kappa_{1}}{\Gamma \kappa_{1}+1}-\frac{\Gamma }{\kappa_{2}(\Gamma +\kappa_{2})}\right]\frac{1}{\lambda (\lambda^{2}-1)}+\frac{1}{\lambda^{3}}\left[\frac{\kappa_{2}+3}{\kappa_{2}(\kappa_{2}+1)}-\frac{1}{\Gamma \kappa_{1}+1}-\frac{3\Gamma }{\kappa_{2}(\Gamma +\kappa_{2})}\right],$$

which is found equivalent to Equation (7.8) by Dundurs [16] in terms of the two Dundurs parameters *α* and *β*.

## 4. Conclusion

We have solved the interaction problem between an edge dislocation and a circular elastic inhomogeneity with Steigmann–Ogden interface. Using analytic continuation, the satisfaction of the continuity condition of displacements across the circular interface leads to the expressions for $\varphi_{2}(z)$ and $\psi_{2}(z)$ in terms of $\varphi_{1}(z)$ and $\psi_{1}(z)$ in Equation (10). Once $\varphi_{1}(z)$ and $\psi_{1}(z)$ are expanded in Taylor series in Equation (13), the complex form of the Steigmann–Ogden interface condition in Equation (4)_2_ can be expressed explicitly: its left-hand side is given by Equation (15) and its right-hand side by Equation (17). Consequently, all of the unknowns in the Taylor series are uniquely determined in Equations (18)–(20) and $\varphi_{2}(z)$ and $\psi_{2}(z)$ are then given explicitly in Equation (25). A general expression for the image force acting on the edge dislocation is given by Equation (26), and the explicit expression of the image force on a gliding edge dislocation is presented in Equation (29). Finally, we note that the problem of an edge dislocation inside a circular elastic inhomogeneity with Steigmann-Ogden interface can be solved in a similar manner.
